# Editorial: Genome-wide molecular mechanisms of substance use disorders

**DOI:** 10.3389/fpsyt.2023.1356103

**Published:** 2024-01-08

**Authors:** Ilya O. Blokhin, David M. Martinez-Garza, Dhruti Patel, Antoine B. Douaihy, Ihsan M. Salloum

**Affiliations:** ^1^Department of Psychiatry and Behavioral Sciences, University of Miami, Miami, FL, United States; ^2^Deparment of Psychiatry, Harvard University, Boston, MA, United States; ^3^Department of Psychiatry, University of Pittsburgh, Pittsburgh, PA, United States; ^4^Department of Neuroscience, University of Texas Rio Grande Valley, Edinburg, TX, United States

**Keywords:** alcohol use disorder, cannabis use disorder, genome-wide association studies, interactome, small interfering RNA, CRISPR-Cas9

Substance use disorders (SUDs) represent significant medical and socioeconomic problem. In the United States, while the opioid epidemic received heightened attention in recent years, the prevalence of other SUDs, particularly alcohol use disorder (AUD) and cannabis use disorder (CaUD), rises, reaching epidemic proportions as well (1). 14% of adults meet criteria for AUD and 29% met AUD criteria at least once during their lifetime (2). Prevalence of CaUD also increases, likely reflecting the legalization of marijuana across multiple states, and has reached 1.23% among adults (3). Current pharmacological options for AUD and CaUD are limited. AUD is typically treated with either naltrexone or acamprosate. These medications are moderately effective (4, 5), and relapse rates in AUD remain ~70–80% (6, 7). There are currently no FDA-approved medications for CaUD.

Lack of efficient treatment modalities often indicates insufficient insight into etiopathogenesis and molecular mechanisms of SUDs as well as anatomical regions and neuro-circuitries involved in the progression of these conditions. Although we begin to appreciate the complexity of addiction-induced global changes across brain regions, treatment strategies still revolve around single target (opioid receptors for naltrexone and NMDA receptors for acamprosate). The concept of the “silver bullet” has been generally accepted in likely most medical specialties, apparently emerging as a consequence of most successful treatments historically being developed for diseases with a clearly defined single pathogenetic mechanism (insulin for type 1 diabetes mellitus, imatinib for chronic myelogenous leukemia) and technical difficulties in targeting multiple pathways simultaneously. In the realm of internal medicine, this concept has nevertheless been successful also in the settings of multifactorial conditions, as evidenced by the utility of angiotensin-converting enzyme inhibitors or angiotensin receptor blockers for essential hypertension and statins for hyperlipidemia (even though treatment algorithms in these conditions continue to rely mainly on trial-and-error approach and many patients are resistant to first-line medicines (8)). Most mental health disorders are the result of complex interactions between numerous biological, environmental, and social determinants, likely far more complicated than in somatic diseases, and the concept of “silver bullet” has been particularly difficult to implement, and relapse rates in psychiatric diseases including SUDs are much higher than in non-psychiatric illnesses (9, 10).

It is currently clear that pathogenesis of addiction involves hundreds of genes and transcripts, with impairment of fundamental genome-wide molecular processes, but there are several important questions which have to be addressed. First, most studies are based on large sample sizes and therefore detect genes/transcripts commonly involved in SUDs, failing to identify rare variants responsible for pathogenesis in specific subpopulations which, in turn, hampers the development of personalized treatment approaches. Another obstacle is that investigations typically focus on one layer of informational flow (genome, transcriptome, or proteome) which provides limited insight into how the whole interactome is affected. As an example, genome-wide association studies have not been able to provide a comprehensive insight into etiopathogenesis of SUDs (11, 12), suggesting that more extensive studies of posttranscriptional mechanisms coupled with subsequent integration of genetic and epigenetic datasets as well as proteome and metabolome may be required.

The current Research Topic is an effort to further highlight the molecular complexity of addiction, using AUD and CaUD as examples. Study by Hill and Hostyk discerned new genetic loci associated with AUD in specific populations. Authors performed the analysis of multiplex families with AUD and detected a distinct, ultra-rare loss-of-function genes implicated in AUD, suggesting novel therapeutic targets specific for these patients. Another interesting AUD study was performed by Zhang et al.; while most investigations focus on genome and transcriptome, authors used liquid chromatography-mass spectrometry to profile serum metabolome in patients with AUD, identifying specific metabolomic profiles which may serve as biomarkers or/and represent pathogenetic links mediating systemic effects of AUD. Reece and Hulse (a, b) have embarked on a comprehensive assessment of dysregulation of interactome in the settings of CaUD, with an emphasis on epigenome, metabolome, immunome, and their interconnectedness. Of note, striking similarity was found between global molecular effects of cannabis and changes which accompany/mediate the process of aging.

These studies indicate that there are multiple knowledge gaps and that more work is needed to build the interactome in relevant brain regions and characterize its impairment in SUDs. Once addiction-related “patho-interactome” has been developed, new set of studies will be required to understand how it can be “repaired”. It is possible that such molecules as transcription factors and non-coding RNAs, being functionally pleiotropic (including their ability to interact simultaneously with proteins and RNAs), may potentially serve as “molecular corkscrews”. Their targeting may be achieved with either small molecules or nucleotide-based therapeutics. CRISPR-Cas9, for instance, offers a simple approach to make changes in genetic code. At mRNA levels, several tools for manipulation have been available for decades, with major technologies represented by small interfering RNAs (siRNAs), antisense oligonucleotides (ASOs), and morpholinos. Manipulation of genome and transcriptome on a genome-wide scale is getting increasingly feasible. Multiplexing editing of mammalian genome using CRISPR/Cas system was shown as early as in 2013 (13) and since then has been replicated multiple times (14). Multiplexing siRNAs and ASOs is more challenging because both technologies function only with support of enzymatic complexes, but morpholinos act via “steric blocking” and do not require intracellular machineries. Delivery of new medicines in the brain will represent another challenge. One potential approach is to use ultrasound-responsive nanoparticles which would release loaded medications in a specific brain region. In this regard, however, another layer of complexity should be taken into consideration. Gene expression profiles are highly dependent on the cellular lineage. For instance, transcriptomic effects of alcohol were distinctly different in astrocytes and microglia (15, 16). Delivery may therefore rather be executed based on cellular origin than anatomical site; in this case, exosomes loaded with therapeutics and expressing complementary epitope/protein (17) could represent one possible technology.

## Author contributions

IB: Writing – original draft, Writing – review & editing. DM-G: Writing – review & editing. DP: Writing – review & editing. AD: Writing – review & editing. IS: Writing – review & editing.
